# Supplementary material for: epDevAtlas: mapping GABAergic cells and microglia in the early postnatal mouse brain
Source: Nat Commun. 2025 Oct 29;16:9538. doi: 10.1038/s41467-025-64549-x (PMC12572312; doi:10.1038/s41467-025-64549-x)
Supplement: Supplementary file 1 — Supplementary info [file 41467_2025_64549_MOESM1_ESM.pdf]

Supplementary Information

Supplementary Figure 1. MRI validation of STPT-based epDevAtlas brain volumes

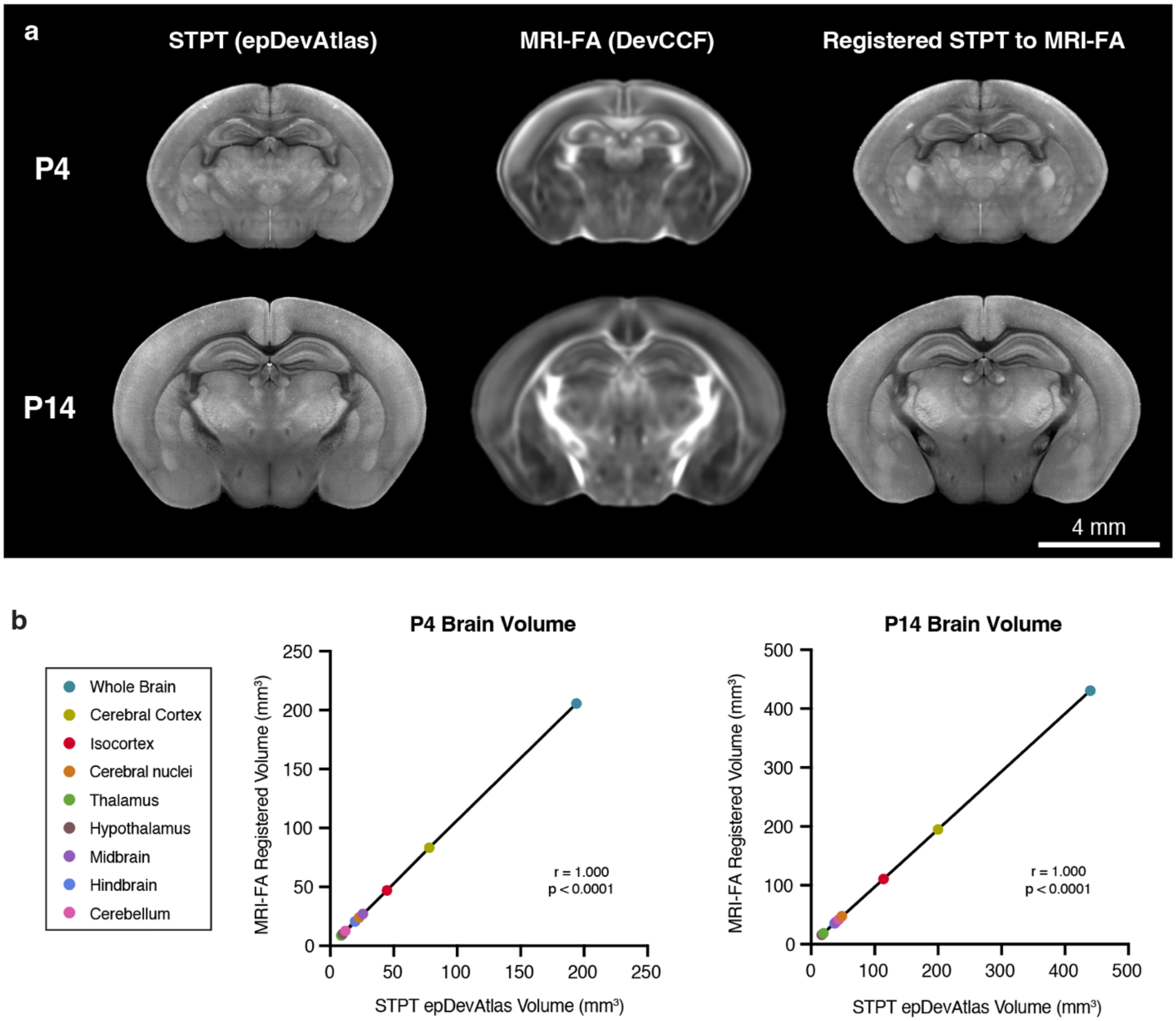

a, Averaged images of P4 (top row) and P14 (bottom row) early postnatal mouse brains acquired by STPT (epDevAtlas, P4: n=5, P14: n=6) and MRI-FA (fractional anisotropy, P4: n=10, P14: n=14) (DevCCF, middle column). Registered STPT-to-MRI-FA images (right column). **b,** Major brain region volume measurements from DevCCF MRI scans registered to our STPT templates at P4 and P14 showed high Pearson correlation coefficients ($r = 1.000$, $p < 0.0001$) Note: Correlation graphs plot the native STPT-acquired volumes from epDevAtlas (x-axis) against the registered STPT-to-MRI volumes in MRI space (y-axis). Source data are provided as a Source Data file.

Supplementary Figure 2. Gene expression changes of early postnatal GABAergic subclasses

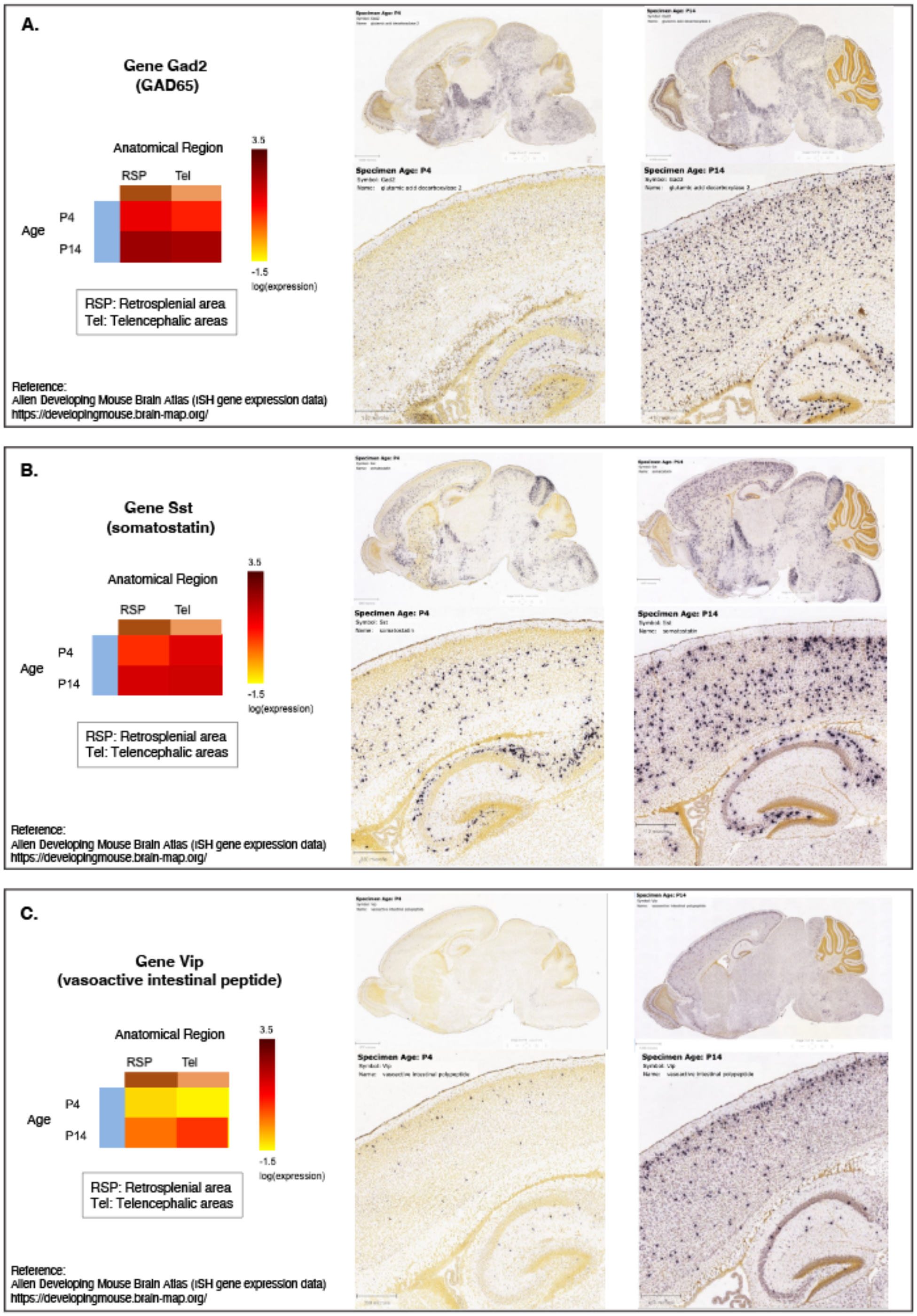

a-c, In situ hybridization (ISH) data from the Allen Developing Mouse Brain Atlas (ADMBA) for *Gad2* (**a**), *Sst* (**b**), and *Vip* (**c**) at P4 and P14. Across telencephalic (Tel) and retrosplenial (RSP) cortical regions, the ISH data for *Gad2, Vip*, and *Sst* show an elevation of gene expression from P4 to P14.

Supplementary Figure 3. Cortical layer developmental mapping of *Gad2, Sst*, and *Vip* neurons

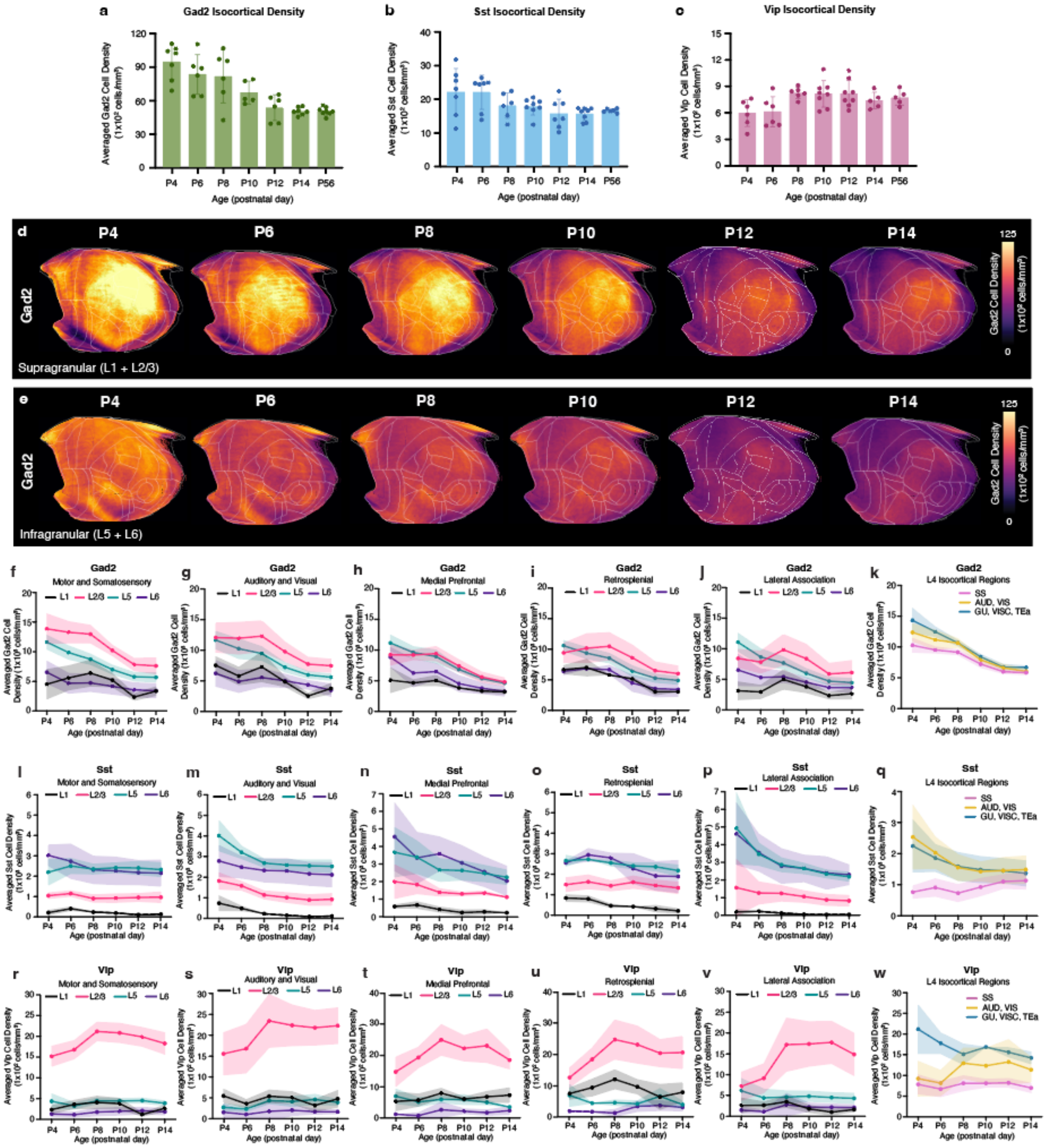

a-c, Averaged GABAergic interneuron subclass densities from P4 to P56: *Gad2* (a), *Sst* (b), and *Vip* (c). **d-e**. Isocortical flatmaps of *Gad2* neuronal densities in **(d)** supragranular (L1, L2/3, L4) and **(e)** infragranular (L5, L6) cortical layers. **f-k,** Layer-specific trajectories of *Gad2* cell density in L1, L2/3, L5, and L6 of the isocortex divided into regional subgroups based on their functional and anatomical connectivity: **(f)** motor and somatosensory, **(g)** auditory and visual, **(h)** medial prefrontal, **(i)** retrosplenial, and **(j)** lateral association areas. **k,** *Gad2* cell density in

isocortical regions containing L4, which includes somatosensory (SS), auditory (AUD), visual (VIS), gustatory (GU), visceral (VISC), and temporal association (TEa) areas. **l-q,** Layer-specific trajectories of isocortical *Sst* cell density. **r-w,** Layer-specific trajectories of *Vip* cell density in the isocortical areas. All data are reported as mean ± s.d. (shaded area between error bars). Standard deviation for layer-specific plots is for the mean densities from each region (ie. SSp-bfd: primary somatosensory, barrel field region) included within the larger regional domain (ie. motor and somatosensory). See Source Data for cell counts, density, and volume measurements for *Gad2, Sst*, and *Vip* neurons, all of which are provided as a Source Data file.

Supplementary Figure 4. Morphological characterization and cortical layer developmental mapping of microglia

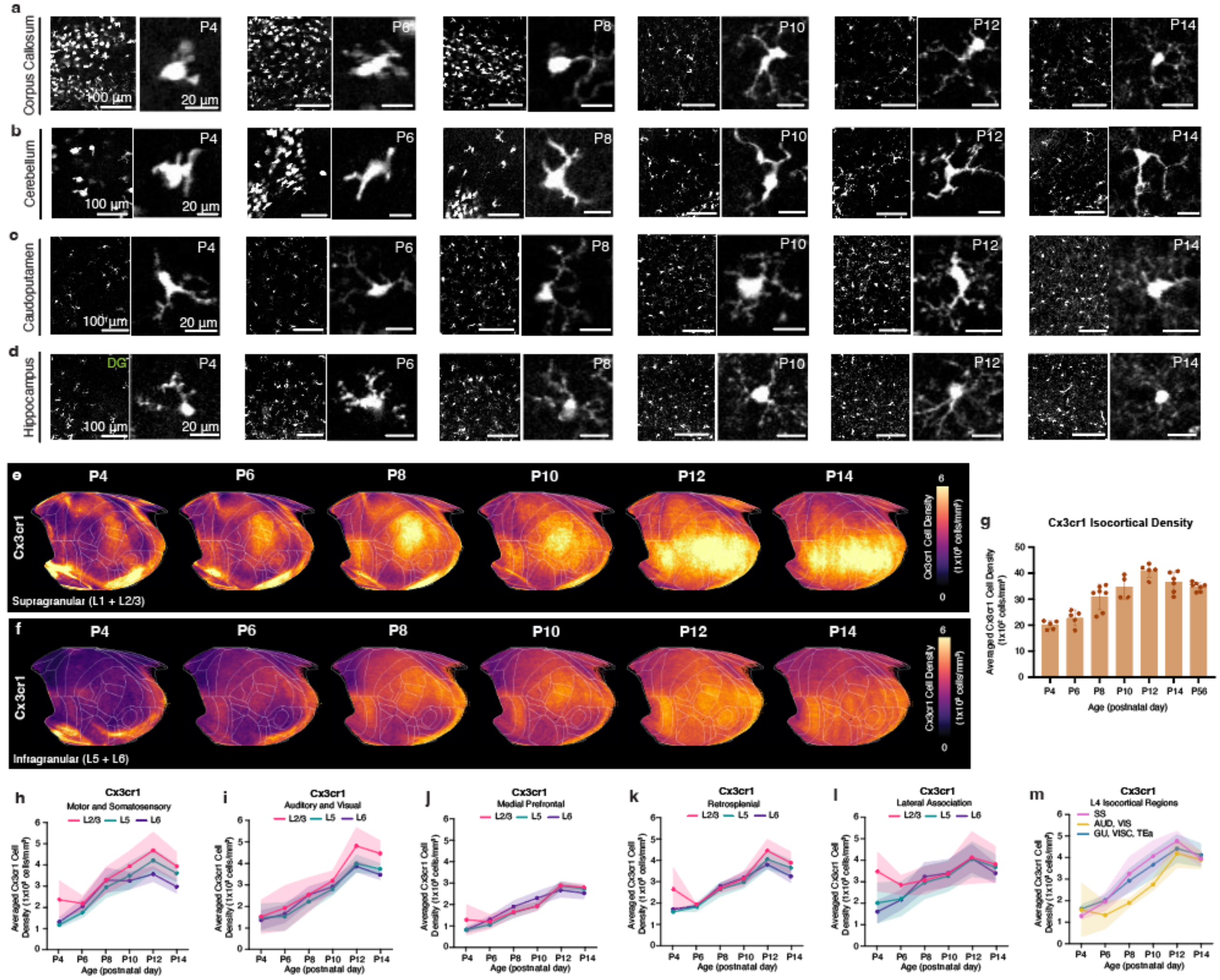

a-d, Representative STPT images of *Cx3cr1* microglia in various brain regions at P4, 6, 8, 10, and 14. Low (left) and high magnification (right) images from each age. (**a**) Amoeboid-shaped, white matter tract-associated microglia (WAMs) with large somas and short, thick branches are present in the corpus callosum from P4 until P8, before adopting a more ramified morphology with longer, extended processes at P10 and onward. (**b**) Likewise, these WAMs with similar morphological specifications outlined in (**a**) are present in the cerebellar white matter. Microglia in gray matter brain regions, such as the (**c**) caudoputamen and the (**d**) dentate gyrus (DG) of the hippocampus exhibit different morphological changes compared to WAMs, with smaller somas and short processes that become increasingly larger and longer, respectively. **e-f,** Isocortical flatmaps of *Cx3cr1* microglial densities ranging from P4 to P14, showing the distinct spatial distribution patterns between **(e)** supragranular (L1, L2/3, L4) and **(f)** infragranular (L5, L6) cortical layers. **g,** Averaged *Cx3cr1* microglial density trend from P4 to P56. **h-m**, Layer-specific trajectories of averaged *Cx3cr1* microglial density in L1, L2/3, L5, and L6 of the isocortex divided into regional subgroups based on their functional and anatomical connectivity: (**h**) motor and somatosensory, (**i**) auditory and visual, (**j**) medial prefrontal, (**k**) retrosplenial, (**l**) lateral

association areas, **(m)** isocortical regions containing L4. All data are reported as mean ± s.d. (shaded area between error bars). See Source Data for *Cx3cr1* microglia count, density, and volume measurements, all of which are provided as a Source Data file.

Supplementary Figure 5. Functional data analysis (FDA) of isocortical cell type densities

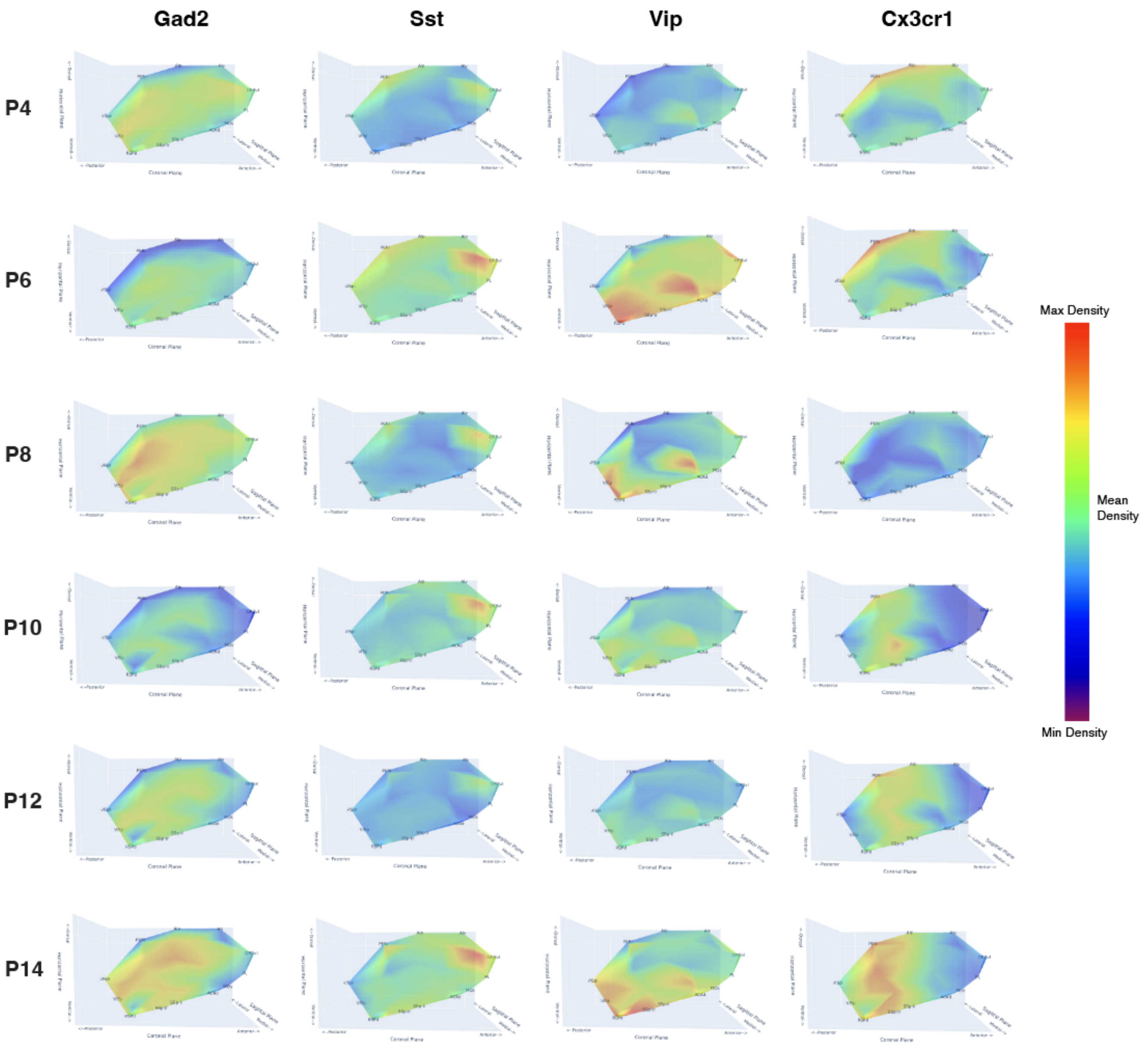

This visualization highlights age-related spatial patterns in density topographies across the isocortex. Each individual panel shows the centered and scaled density distribution for one cell type at a specific postnatal age (P4–P14), with cell types arranged in columns and ages in rows. Density values are centered around the mean (0) and displayed on a rainbow color scale, with the color midpoint of green representing mean density, blue for minimum (min) density, and red for maximum (max) density.

Supplementary Figure 6. Pairwise differences in isocortical cell density topographies across age groups for *Cx3cr1* microglia and *Sst* interneurons

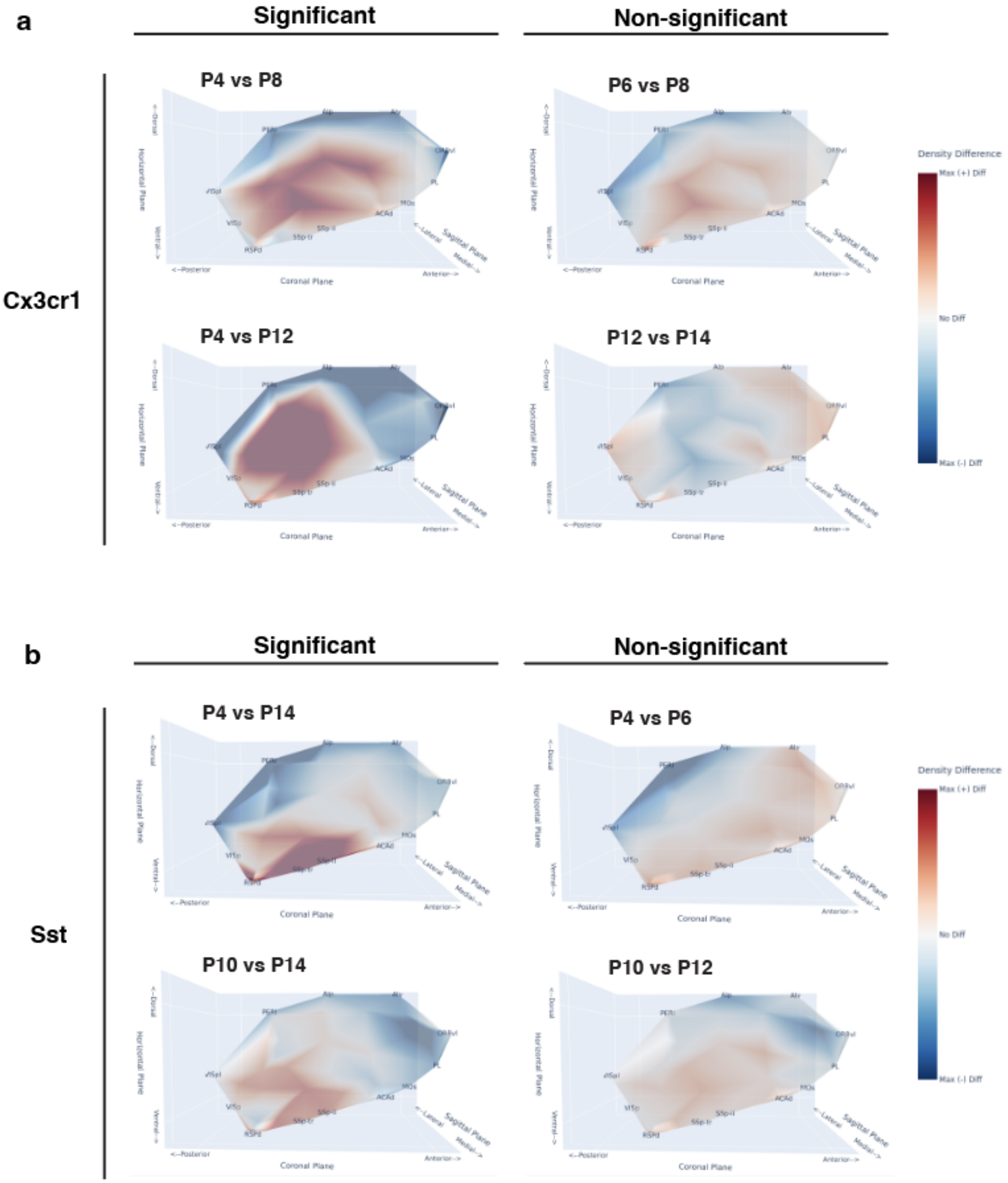

a-b. Panels show age-wise differences in density topographies for (**a**) *Cx3cr1* microglia and (**b**) *Sst* interneurons, for which permutation ANOVA on reconstruction errors across all ages was significant. Pairwise comparisons with significant differences (first column) are highlighted alongside example pairs without significant differences (second column) for comparison of density topographies. Differences are displayed on a red-blue color scale, where red indicates positive differences and blue indicates negative differences in cell density topographies.

Supplementary Figure 7. Elastix image registration parameters

Rigid elastix parameters:

```
// Example parameter file for rotation registration
// C-style comments: //

// The internal pixel type, used for internal computations
// Leave to float in general.
// NB: this is not the type of the input images! The pixel
// type of the input images is automatically read from the
// images themselves.
// This setting can be changed to "short" to save some memory
// in case of very large 3D images.
(FixedInternalImagePixelType "float")
(MovingInternalImagePixelType "float")

// The dimensions of the fixed and moving image
// Up to elastix 4.5 this had to be specified by the user.
// From elastix 4.6, this is not necessary anymore.
//(FixedImageDimension 2)
//(MovingImageDimension 2)

// Specify whether you want to take into account the so-called
// direction cosines of the images. Recommended: true.
// In some cases, the direction cosines of the image are corrupt,
// due to image format conversions for example. In that case, you
// may want to set this option to "false".
(UseDirectionCosines "true")

// **************** Main Components **************************

// The following components should usually be left as they are:
(Registration "MultiResolutionRegistration")
(Interpolator "BSplineInterpolator")
(ResampleInterpolator "FinalBSplineInterpolator")
(Resampler "DefaultResampler")

// These may be changed to Fixed/MovingSmoothingImagePyramid.
// See the manual.
(FixedImagePyramid "FixedRecursiveImagePyramid")
(MovingImagePyramid "MovingRecursiveImagePyramid")

// The following components are most important:
// The optimizer AdaptiveStochasticGradientDescent (ASGD) works
// quite ok in general. The Transform and Metric are important
```

```
// and need to be chosen careful for each application. See manual.
(Optimizer "AdaptiveStochasticGradientDescent")
(Transform "SimilarityTransform")
(Metric "AdvancedMattesMutualInformation")

// ***************** Transformation **************************

// Scales the rotations compared to the translations, to make
// sure they are in the same range. In general, it's best to
// use automatic scales estimation:
(AutomaticScalesEstimation "true")

// Automatically guess an initial translation by aligning the
// geometric centers of the fixed and moving.
(AutomaticTransformInitialization "true")

// Whether transforms are combined by composition or by addition.
// In generally, Compose is the best option in most cases.
// It does not influence the results very much.
(HowToCombineTransforms "Compose")

// ******************* Similarity measure *********************

// Number of grey level bins in each resolution level,
// for the mutual information. 16 or 32 usually works fine.
// You could also employ a hierarchical strategy:
//(NumberOfHistogramBins 16 32 64)
(NumberOfHistogramBins 32)

// If you use a mask, this option is important.
// If the mask serves as region of interest, set it to false.
// If the mask indicates which pixels are valid, then set it to true.
// If you do not use a mask, the option doesn't matter.
(ErodeMask "false")

// ******************** Multiresolution **********************

// The number of resolutions. 1 Is only enough if the expected
// deformations are small. 3 or 4 mostly works fine. For large
// images and large deformations, 5 or 6 may even be useful.
(NumberOfResolutions 4)

// The downsampling/blurring factors for the image pyramids.
// By default, the images are downsampled by a factor of 2
// compared to the next resolution.
// So, in 2D, with 4 resolutions, the following schedule is used:
```

```
//(ImagePyramidSchedule 8 8  4 4  2 2  1 1 )
// And in 3D:
//(ImagePyramidSchedule 8 8 8  4 4 4  2 2 2  1 1 1 )
// You can specify any schedule, for example:
//(ImagePyramidSchedule 4 4  4 3  2 1  1 1 )
// Make sure that the number of elements equals the number
// of resolutions times the image dimension.

// ****************** Optimizer ****************************

// Maximum number of iterations in each resolution level:
// 200-500 works usually fine for rigid registration.
// For more robustness, you may increase this to 1000-2000.
(MaximumNumberOfIterations 2000)

// The step size of the optimizer, in mm. By default the voxel size is used.
// which usually works well. In case of unusual high-resolution images
// (eg histology) it is necessary to increase this value a bit, to the size
// of the "smallest visible structure" in the image:
//(MaximumStepLength 1.0)

// **************** Image sampling **********************

// Number of spatial samples used to compute the mutual
// information (and its derivative) in each iteration.
// With an AdaptiveStochasticGradientDescent optimizer,
// in combination with the two options below, around 2000
// samples may already suffice.
(NumberOfSpatialSamples 2048)

// Refresh these spatial samples in every iteration, and select
// them randomly. See the manual for information on other sampling
// strategies.
(NewSamplesEveryIteration "true")
(ImageSampler "Random")

// ************* Interpolation and Resampling ****************

// Order of B-Spline interpolation used during registration/optimisation.
// It may improve accuracy if you set this to 3. Never use 0.
// An order of 1 gives linear interpolation. This is in most
// applications a good choice.
(BSplineInterpolationOrder 1)

// Order of B-Spline interpolation used for applying the final
// deformation.
```

// 3 gives good accuracy; recommended in most cases.
// 1 gives worse accuracy (linear interpolation)
// 0 gives worst accuracy, but is appropriate for binary images
// (masks, segmentations); equivalent to nearest neighbor interpolation.
(FinalBSplineInterpolationOrder 0)

//Default pixel value for pixels that come from outside the picture:
(DefaultPixelValue 0)

// Choose whether to generate the deformed moving image.
// You can save some time by setting this to false, if you are
// only interested in the final (nonrigidly) deformed moving image
// for example.
(WriteResultImage "false")

// The pixel type and format of the resulting deformed moving image
(ResultImagePixelType "float")
(ResultImageFormat "mhd")

B-spline elastix parameters:

// Example parameter file for B-spline registration
// C-style comments: //

// The internal pixel type, used for internal computations
// Leave to float in general.
// NB: this is not the type of the input images! The pixel
// type of the input images is automatically read from the
// images themselves.
// This setting can be changed to "short" to save some memory
// in case of very large 3D images.
(FixedInternalImagePixelType "float")
(MovingInternalImagePixelType "float")

// The dimensions of the fixed and moving image
// Up to elastix 4.5 this had to be specified by the user.
// From elastix 4.6, this is not necessary anymore.
//(FixedImageDimension 2)
//(MovingImageDimension 2)

// Specify whether you want to take into account the so-called
// direction cosines of the images. Recommended: true.
// In some cases, the direction cosines of the image are corrupt,
// due to image format conversions for example. In that case, you

```
// may want to set this option to "false".
(UseDirectionCosines "true")

// **************** Main Components **************************

// The following components should usually be left as they are:
(Registration "MultiResolutionRegistration")
(Interpolator "BSplineInterpolator")
(ResampleInterpolator "FinalBSplineInterpolator")
(Resampler "DefaultResampler")

// These may be changed to Fixed/MovingSmoothingImagePyramid.
// See the manual.
(FixedImagePyramid "FixedRecursiveImagePyramid")
(MovingImagePyramid "MovingRecursiveImagePyramid")

// The following components are most important:
// The optimizer AdaptiveStochasticGradientDescent (ASGD) works
// quite ok in general. The Transform and Metric are important
// and need to be chosen careful for each application. See manual.
(Optimizer "AdaptiveStochasticGradientDescent")
(Transform "BSplineTransform")
(Metric "AdvancedMattesMutualInformation")

// ***************** Transformation **************************

// The control point spacing of the bspline transformation in
// the finest resolution level. Can be specified for each
// dimension differently. Unit: mm.
// The lower this value, the more flexible the deformation.
// Low values may improve the accuracy, but may also cause
// unrealistic deformations. This is a very important setting!
// We recommend tuning it for every specific application. It is
// difficult to come up with a good 'default' value.
//(FinalGridSpacingInPhysicalUnits 64)

// Alternatively, the grid spacing can be specified in voxel units.
// To do that, uncomment the following line and comment/remove
// the FinalGridSpacingInPhysicalUnits definition.
(FinalGridSpacingInVoxels 400)

// By default the grid spacing is halved after every resolution,
// such that the final grid spacing is obtained in the last
// resolution level. You can also specify your own schedule,
// if you uncomment the following line:
//(GridSpacingSchedule 4.0 4.0 2.0 1.0)
```

```
// This setting can also be supplied per dimension.

// Whether transforms are combined by composition or by addition.
// In generally, Compose is the best option in most cases.
// It does not influence the results very much.
(HowToCombineTransforms "Compose")

// ****************** Similarity measure ********************

// Number of grey level bins in each resolution level,
// for the mutual information. 16 or 32 usually works fine.
// You could also employ a hierarchical strategy:
//(NumberOfHistogramBins 16 32 64)
(NumberOfHistogramBins 64)

// If you use a mask, this option is important.
// If the mask serves as region of interest, set it to false.
// If the mask indicates which pixels are valid, then set it to true.
// If you do not use a mask, the option doesn't matter.
(ErodeMask "false")

// ******************* Multiresolution *********************

// The number of resolutions. 1 Is only enough if the expected
// deformations are small. 3 or 4 mostly works fine. For large
// images and large deformations, 5 or 6 may even be useful.
(NumberOfResolutions 6)

// The downsampling/blurring factors for the image pyramids.
// By default, the images are downsampled by a factor of 2
// compared to the next resolution.
// So, in 2D, with 4 resolutions, the following schedule is used:
//(ImagePyramidSchedule 8 8  4 4  2 2  1 1 )
// And in 3D:
//(ImagePyramidSchedule 8 8 8  4 4 4  2 2 2  1 1 1 )
// You can specify any schedule, for example:
//(ImagePyramidSchedule 4 4  4 3  2 1  1 1 )
// Make sure that the number of elements equals the number
// of resolutions times the image dimension.

// ****************** Optimizer ***************************

// Maximum number of iterations in each resolution level:
// 200-2000 works usually fine for nonrigid registration.
// The more, the better, but the longer computation time.
// This is an important parameter!
```

```
(MaximumNumberOfIterations 2000)

// The step size of the optimizer, in mm. By default the voxel size is used.
// which usually works well. In case of unusual high-resolution images
// (eg histology) it is necessary to increase this value a bit, to the size
// of the "smallest visible structure" in the image:
(MaximumStepLength 4.0)

// **************** Image sampling **********************

// Number of spatial samples used to compute the mutual
// information (and its derivative) in each iteration.
// With an AdaptiveStochasticGradientDescent optimizer,
// in combination with the two options below, around 2000
// samples may already suffice.
(NumberOfSpatialSamples 4096)

// Refresh these spatial samples in every iteration, and select
// them randomly. See the manual for information on other sampling
// strategies.
(NewSamplesEveryIteration "true")
(ImageSampler "Random")

// ************* Interpolation and Resampling ****************

// Order of B-Spline interpolation used during registration/optimisation.
// It may improve accuracy if you set this to 3. Never use 0.
// An order of 1 gives linear interpolation. This is in most
// applications a good choice.
(BSplineInterpolationOrder 1)

// Order of B-Spline interpolation used for applying the final
// deformation.
// 3 gives good accuracy; recommended in most cases.
// 1 gives worse accuracy (linear interpolation)
// 0 gives worst accuracy, but is appropriate for binary images
// (masks, segmentations); equivalent to nearest neighbor interpolation.
(FinalBSplineInterpolationOrder 0)

//Default pixel value for pixels that come from outside the picture:
(DefaultPixelValue 0)

// Choose whether to generate the deformed moving image.
// You can save some time by setting this to false, if you are
// not interested in the final deformed moving image, but only
// want to analyze the deformation field for example.
```

(WriteResultImage "true")

// The pixel type and format of the resulting deformed moving image
(ResultImagePixelType "float")
(ResultImageFormat "mhd")

Supplementary Figure 8. ANTs image registration parameters

```
### paramters for antsRegistration \
--verbose 1 \
--dimensionality 3 \
--float 1 \
--collapse-output-transforms 1 \
--interpolation Bspline \
--use-histogram-matching 1 \
--winsorize-image-intensities [ 0.005,0.995 ] \
--transform Rigid[ 0.1 ] \
   --metric MI[ ${fixedImage0},${movingImage0},1,32,Regular,0.25 ] \
   --convergence [ 1000x500,1e-6,10 ] \
   --shrink-factors 12x8 \
   --smoothing-sigmas 4x3vox \
--transform Affine[ 0.1 ] \
   --metric MI[ ${fixedImage0},${movingImage0},1,32,Regular,0.25 ] \
   --convergence [ 1000x500x200,1e-6,10 ] \
   --shrink-factors 10x8x6 \
   --smoothing-sigmas 5x4x3vox \
--transform SyN[ 0.1,3,0 ] \
   --metric CC[ ${fixedImage0},${movingImage0},0.4,4] \
   --metric CC[ ${fixedImage1},${movingImage1},0.3,4] \
   --metric CC[ ${fixedImage2},${movingImage2},0.3,4] \
   --convergence [ 200x100x100,1e-6,10 ] \
   --shrink-factors 10x6x4 \
   --smoothing-sigmas 5x3x2vox


### paramters for antsApplyTransforms
-d 3 -v 1 \
-i $transformFileLabel \
-r $fixedImage0 \
-o $transform_output_name_label \
-n MultiLabel \
-t ${outputPrefix}1Warp.nii.gz \
-t ${outputPrefix}0GenericAffine.mat


### parameters for landmark antsRegistration
--verbose 1 \
--dimensionality 3 \
--float 1 \
--collapse-output-transforms 1 \
--output [ ${outputPrefix},${outputPrefix}warped.nii.gz,${outputPrefix}inverseWarped.nii.gz ]
\
```

```
--interpolation Bspline \
--transform SyN[ 0.1,3,0 ] \
    --metric MeanSquares[ ${fixedImage0},${movingImage0},1,0] \
    --convergence [ 100x100,1e-4,10 ] \
    --shrink-factors 4x2 \
    --smoothing-sigmas 2x1vox


### paramters for landmark antsApplyTransforms
-d 3 -v 1 \
-i $transformFileLabel \
-r $fixedImage0 \
-o $transform_output_name_label \
-n MultiLabel \
-t ${outputPrefix}0Warp.nii.gz
```

Supplementary Table 1. Driver and Reporter Mouse Lines Used for Cell Type Labeling

#	Line Name	Gene	Driver Mouse Line	Reporter Mouse Line	Cell Type Labeled and Considered for Study
1	Gad2-IRES-Cre; Ai14	Gad2	Gad2-IRES-Cre	Ai14 (tdTomato)	Pan-GABAergic (Gad2-expressing) neurons
2	Sst-IRES-Cre; Ai14	Sst	Sst-IRES-Cre	Ai14 (tdTomato)	Somatostatin (Sst-expressing) neurons
3	Vip-IRES-Cre; Ai14	Vip	Vip-IRES-Cre	Ai14 (tdTomato)	Vasoactive intestinal peptide (Vip-expressing) neurons
5	Slc32a1-IRES-Cre; Lamp5-P2A-FlpO; Ai65	Slc32a1/ Lamp5	Slc32a1-IRES-Cre;Lamp5-P2A-FlpO	Ai65 (tdTomato)	Slc32a1/Lamp5-expressing Layer 1 cortical neurons
6	Calb2-IRES-Cre; Ai14	Calb2	Calb2-IRES-Cre	Ai14 (tdTomato)	Calb2-expressing Layer 2/3 cortical neurons
7	Nr5a1-Cre; Ai14	Nr5a1	Nr5a1-Cre	Ai14 (tdTomato)	Nr5a1-expressing Layer 4 cortical neurons
8	Rbp4-Cre_KL100; Ai14	Rbp4	Rbp4-Cre_KL100	Ai14 (tdTomato)	Rbp4-expressing Layer 5 cortical neurons
9	Ntsr1-Cre_GN220; Ai14	Ntsr1	Ntsr1-Cre_GN220	Ai14 (tdTomato)	Ntsr1-expressing Layer 6 cortical neurons
10	Cplx3-P2A-FlpO; Ai193	Cplx3	Cplx3-P2A-FlpO	Ai193 (Flp: tdTomato)	Cplx3-expressing Layer 6b cortical neurons
11	Cx3cr1-GFP(+/-)	Cx3cr1	Cx3cr1-GFP (+/-)	-	Cx3cr1-eGFP-expressing brain microglia

Note that some transgenic lines are not exclusively expressed but strongly enriched in certain cell types.

Supplementary Table 2. Sample Size, Age, and Sex of Study Animals

	Number of Animals per Age												Total number
**Age**	**P4**		**P6**		**P8**		**P10**		**P12**		**P14**		
**Sex**	**M**	**F**	**M**	**F**	**M**	**F**	**M**	**F**	**M**	**F**	**M**	**F**	
Gad2-IRES-Cre; Ai14	3	4	3	3	3	3	3	2	2	4	3	4	37
Sst-IRES-Cre; Ai14	4	3	4	3	3	3	5	3	3	4	4	4	43
Vip-IRES-Cre; Ai14	3	4	4	2	3	3	4	4	4	4	3	4	42
Slc32a1-IRES-Cre; Lamp5-P2A-FlpO; Ai65	-	-	1	1	-	-	1	1	-	-	1	1	6
Calb2-IRES-Cre; Ai14	-	-	1	1	-	-	1	1	-	-	-	2	6
Nr5a1-Cre; Ai14	-	-		2	-	-	1	1	-	-	2	-	6
Rbp4-Cre_KL100; Ai14	-	-	1	1	-	-	1	1	-	-	2	-	6
Ntsr1-Cre_GN220; Ai14	-	-	1	1	-	-	2		-	-	2	-	6
Cplx3-P2A-FlpO; Ai193	-	-	1	1	-	-	1	1	-	-	1	1	6
Cx3cr1-GFP(+/-)	2	3	3	2	3	4	1	3	2	3	2	4	32
												All:	190

Supplementary Table 3. Additional Driver Line Strain Information

#	Line Name	Abbreviation	Lab or Investigator of Origin	Primary Reference	Generation Method	RRID	Public Repository & Stock #	Repository Strain Name
1	Gad2-IRES-Cre	Gad2	Z. Josh Huang and Melissa Warden	Taniguchi et al., Neuron 2011	Knock-in (IRES)	RRID:IMSR_JAX:028867	The Jackson Laboratory (028867)	B6J.Cg-$\mathit{Gad2}^{tm2(cre)Zjh}$/MwarJ
2	Sst-IRES-Cre	Sst	Z. Josh Huang	Taniguchi et al., Neuron 2011	Knock-in (IRES)	RRID:IMSR_JAX:013044	The Jackson Laboratory (013044)	STOCK $\mathit{Sst}^{tm2.1(cre)Zjh}$/J
3	Vip-IRES-Cre	Vip	Z. Josh Huang and Andrew Recknagel	Taniguchi et al., Neuron 2011	Knock-in (IRES)	RRID:IMSR_JAX:031628	The Jackson Laboratory (031628)	B6J.Cg-$\mathit{Vip}^{tm1(cre)Zjh}$/AreckJ
4	Pvalb-IRES-Cre	Pvalb	Silvia Arber	Hippenmeyer et al., PLoS Biol 2005	Knock-in (IRES)	RRID:IMSR_JAX:017320	The Jackson Laboratory (017320)	B6.129P2-$\mathit{Pvalb}^{tm1(cre)Arbr}$/J
5	Lamp5-P2A-FlpO	Lamp5	John Ngai and Bosiljka Tasic	N/A	Knock-in (P2A)	RRID:IMSR_JAX:037340	The Jackson Laboratory (037340)	B6.Cg-$\mathit{Lamp5}^{em1(flpo*)Ngai}$/TasicJ
6	Slc32a1-IRES-Cre	Slc32a1	Bradford Lowell	Tong et al., Nat. Neu. 2008	Knock-in (IRES)	RRID:IMSR_JAX:012897	The Jackson Laboratory (012897)	STOCK $\mathit{Slc32a1}^{tm1Lowl}$/J
7	Calb2-IRES-Cre	Calb2	Z. Josh Huang	Taniguchi et al., Neuron 2011	Knock-in (IRES)	RRID:IMSR_JAX:010774	The Jackson Laboratory (010774)	B6(Cg)-$\mathit{Calb2}^{tm1(cre)Zjh}$/J
8	Nr5a1-Cre	Nr5a1	Bradford Lowell	Dhillon et al., Neuron 2006	Transgenic (BAC)	RRID:IMSR_JAX:006364	The Jackson Laboratory (006364)	FVB-Tg(Nr5a1-cre)2Lowl/J

9	Rbp4-Cre_KL100	Rbp4	Nathaniel Heintz and Charles Gerfen	Gerfen et al., Neuron 2013	Transgenic (BAC)	RRID: MMRRC_031125-UCD	MMRRC (031125)	STOCK Tg(Rbp4-cre)KL100Gsat/Mmucd
10	Ntsr1-Cre_GN220	Ntsr1	Nathaniel Heintz and Charles Gerfen	Gerfen et al., Neuron 2013	Transgenic (BAC)	RRID: MMRRC_030648-UCD	MMRRC (030648)	B6.FVB(Cg)-Tg(Ntsr1-cre)GN220Gsat/Mmucd
11	Cplx3-P2A-FlpO	Cplx3	John Ngai and Bosiljka Tasic	N/A	Knock-in (P2A)	RRID:IMSR_JAX:037338	The Jackson Laboratory (037338)	B6.Cg-*Cplx3*$^{em1(flpo*)Ngai}$/TasicJ
12	Cx3cr1-GFP	Cx3cr1	Dan Littman	Jung et al., Mol Cell Biol 2000	Knock-in/Knock-out (eGFP site insert)	RRID:IMSR_JAX:005582	The Jackson Laboratory (005582)	B6.129P2(Cg)-*Cx3cr1*^{tm1Litt}/J
13	C57BL/6J	C57BL/6J	CC Little	Multiple sources	Inbred (N/A)	RRID:IMSR_JAX:000664	The Jackson Laboratory (000664)	C57BL/6J

Supplementary Table 4. Additional Reporter Line Strain Information

#	Line Name	Abbreviation	Lab or Investigator of Origin	Primary Reference	Generation Method	RRID	Public Repository & Stock #	Repository Strain Name
1	Ai14(RCL-tdT)	Ai14	Hongkui Zeng, Allen Institute for Brain Science	Madisen et al., Nat. Neu. 2010	Knock-in (N/A)	RRID:IMSR_JAX:007914	The Jackson Laboratory (007914)	B6.Cg-$Gt(ROSA)26Sor^{tm14(CAG-tdTomato)Hze}$/J
2	Ai65(RCFL-tdT)	Ai65	Hongkui Zeng, Allen Institute for Brain Science	Madisen et al., Neuron 2015	Knock-in (N/A)	RRID:IMSR_JAX:021875	The Jackson Laboratory (021875)	B6;129S-$Gt(ROSA)26Sor^{tm65.1(CAG-tdTomato)Hze}$/J
3	Ai193(TICL-EGFP-ICF-tdT)	Ai193	Bosiljka Tasic, Allen Institute for Brain Science	N/A	Knock-in (N/A)	RRID:IMSR_JAX:034111	The Jackson Laboratory (034111)	B6;129S6-$Igs7^{tm193(CAG-EGFP,CAG-tdTomato)Tasic}$/J
