# Supplementary material for: epDevAtlas: mapping GABAergic cells and microglia in the early postnatal mouse brain
Source: Nat Commun. 2025 Oct 29;16:9538. doi: 10.1038/s41467-025-64549-x (PMC12572312; doi:10.1038/s41467-025-64549-x)
Supplement: Supplementary file 2 — Description of Additional Supplementary Files [file 41467_2025_64549_MOESM2_ESM.pdf]

Description of Additional Supplementary Files

Supplementary data 1: Bayesian statistical analysis results. Bayesian multivariate analysis of cell densities with posterior estimates, errors, credible intervals, and diagnostics. Dunnett's T3 post hoc tests provide pairwise group comparisons with mean differences, confidence intervals, and adjusted p-values.

Supplementary data 2: F-score Validation of Cell Counting. F1 score is a harmonic mean of precision and recall (1 being the best and 1 being the poorest). F1 score was calculated separately in each cell type.
