# Supplementary material for: epDevAtlas: mapping GABAergic cells and microglia in the early postnatal mouse brain
Source: Nat Commun. 2025 Oct 29;16:9538. doi: 10.1038/s41467-025-64549-x (PMC12572312; doi:10.1038/s41467-025-64549-x)
Supplement: Supplementary file 4 — Reporting Summary [file 41467_2025_64549_MOESM4_ESM.pdf]

nature portfolio

Corresponding author(s): Yongsoo Kim

Last updated by author(s): Aug 19, 2025

Reporting Summary

Nature Portfolio wishes to improve the reproducibility of the work that we publish. This form provides structure for consistency and transparency in reporting. For further information on Nature Portfolio policies, see our Editorial Policies and the Editorial Policy Checklist.

Statistics

For all statistical analyses, confirm that the following items are present in the figure legend, table legend, main text, or Methods section.

n/a	Confirmed	
☐	☒	The exact sample size (*n*) for each experimental group/condition, given as a discrete number and unit of measurement
☐	☒	A statement on whether measurements were taken from distinct samples or whether the same sample was measured repeatedly
☐	☒	The statistical test(s) used AND whether they are one- or two-sided<br>*Only common tests should be described solely by name; describe more complex techniques in the Methods section.*
☐	☒	A description of all covariates tested
☐	☒	A description of any assumptions or corrections, such as tests of normality and adjustment for multiple comparisons
☐	☒	A full description of the statistical parameters including central tendency (e.g. means) or other basic estimates (e.g. regression coefficient) AND variation (e.g. standard deviation) or associated estimates of uncertainty (e.g. confidence intervals)
☐	☒	For null hypothesis testing, the test statistic (e.g. *F*, *t*, *r*) with confidence intervals, effect sizes, degrees of freedom and *P* value noted<br>*Give P values as exact values whenever suitable.*
☐	☒	For Bayesian analysis, information on the choice of priors and Markov chain Monte Carlo settings
☐	☒	For hierarchical and complex designs, identification of the appropriate level for tests and full reporting of outcomes
☐	☒	Estimates of effect sizes (e.g. Cohen's *d*, Pearson's *r*), indicating how they were calculated

Our web collection on statistics for biologists contains articles on many of the points above.

Software and code

Policy information about availability of computer code

Data collection	Custom code for serial two photon tomography image stitching is available (https://github.com/yongsookimlab)
Data analysis	All custom code created for cell quantification and cortical flatmap generation are available (https://github.com/yongsookimlab)

For manuscripts utilizing custom algorithms or software that are central to the research but not yet described in published literature, software must be made available to editors and reviewers. We strongly encourage code deposition in a community repository (e.g. GitHub). See the Nature Portfolio guidelines for submitting code & software for further information.

Data

Policy information about availability of data

All manuscripts must include a data availability statement. This statement should provide the following information, where applicable:

- Accession codes, unique identifiers, or web links for publicly available datasets
- A description of any restrictions on data availability
- For clinical datasets or third party data, please ensure that the statement adheres to our policy

The epDevAtlas is an openly accessible resource package including age-matched templates and anatomical labels which can be viewed and downloaded via https://kimlab.io/brain-map/epDevAtlas/. Full resolution and mapped cell type data can be also found at https://kimlab.io/brain-map/epDevAtlas/. The data has been deposited in public data repository (Figshare) with DOI: https://doi.org/10.6084/m9.figshare.29910476

Research involving human participants, their data, or biological material

Policy information about studies with human participants or human data. See also policy information about sex, gender (identity/presentation), and sexual orientation and race, ethnicity and racism.

Reporting on sex and gender	*Use the terms sex (biological attribute) and gender (shaped by social and cultural circumstances) carefully in order to avoid confusing both terms. Indicate if findings apply to only one sex or gender; describe whether sex and gender were considered in study design; whether sex and/or gender was determined based on self-reporting or assigned and methods used. Provide in the source data disaggregated sex and gender data, where this information has been collected, and if consent has been obtained for sharing of individual-level data; provide overall numbers in this Reporting Summary. Please state if this information has not been collected. Report sex- and gender-based analyses where performed, justify reasons for lack of sex- and gender-based analysis.*
Reporting on race, ethnicity, or other socially relevant groupings	*Please specify the socially constructed or socially relevant categorization variable(s) used in your manuscript and explain why they were used. Please note that such variables should not be used as proxies for other socially constructed/relevant variables (for example, race or ethnicity should not be used as a proxy for socioeconomic status). Provide clear definitions of the relevant terms used, how they were provided (by the participants/respondents, the researchers, or third parties), and the method(s) used to classify people into the different categories (e.g. self-report, census or administrative data, social media data, etc.) Please provide details about how you controlled for confounding variables in your analyses.*
Population characteristics	*Describe the covariate-relevant population characteristics of the human research participants (e.g. age, genotypic information, past and current diagnosis and treatment categories). If you filled out the behavioural & social sciences study design questions and have nothing to add here, write "See above."*
Recruitment	*Describe how participants were recruited. Outline any potential self-selection bias or other biases that may be present and how these are likely to impact results.*
Ethics oversight	*Identify the organization(s) that approved the study protocol.*

Note that full information on the approval of the study protocol must also be provided in the manuscript.

Field-specific reporting

Please select the one below that is the best fit for your research. If you are not sure, read the appropriate sections before making your selection.

☒ Life sciences ☐ Behavioural & social sciences ☐ Ecological, evolutionary & environmental sciences

For a reference copy of the document with all sections, see nature.com/documents/nr-reporting-summary-flat.pdf

Life sciences study design

All studies must disclose on these points even when the disclosure is negative.

Sample size	The goal of the current study is to provide cell densities from different cell types at different ages rather than comparing differences between experimental groups. In our previous studies (Kim et al., PMID: 28985566; Newmaster et al., PMID: 32313029), we found that cell density variability is relatively stable at a given cell type and age. About five animals were sufficient to provide population density. The total sample size for this study is n=190 animals. For each major cell type (Gad2, Sst, Vip, Cx3cr1), the sample size ranged between n=32 and n=43. Additionally, for each age group per cell type, the sample size was between n=4 and n=8, with approximately equal numbers of males and females.
Data exclusions	Data were excluded if problems occurred during brain sample preparation and imaging that would render missing tissue and imaging artifacts, since this would skew analysis results.
Replication	Each sample underwent the same data collection and analytical pipeline. In this sense, all attempts at replication of the pipeline to produce findings were successful. The reproducibility of the findings were confirmed by repeating the analyses.
Randomization	Each animal was collected and utilized once, as individual animals belonged to a specific group based on postnatal age and genetically-labeled cell type expression. Within each group, mice were randomly selected from different litters, but more than one mouse may be derived from the same litter. To control for litter effects, all group data were averaged before being analyzed for significance.
Blinding	Blinding was not relevant to the study since it did not involve any experimental groups with independent variables and dependent outcomes.

Reporting for specific materials, systems and methods

We require information from authors about some types of materials, experimental systems and methods used in many studies. Here, indicate whether each material, system or method listed is relevant to your study. If you are not sure if a list item applies to your research, read the appropriate section before selecting a response.

Materials & experimental systems

n/a	Involved in the study
☒	☐ Antibodies
☒	☐ Eukaryotic cell lines
☒	☐ Palaeontology and archaeology
☐	☒ Animals and other organisms
☒	☐ Clinical data
☒	☐ Dual use research of concern
☒	☐ Plants

Methods

n/a	Involved in the study
☒	☐ ChIP-seq
☒	☐ Flow cytometry
☒	☐ MRI-based neuroimaging

Animals and other research organisms

Policy information about studies involving animals; ARRIVE guidelines recommended for reporting animal research, and Sex and Gender in Research

Laboratory animals

Laboratory animals used were of the species mus musculus. During early postnatal development at postnatal (P) days P4, P6, P8, P10, P12, and P14, we crossed Gad2-IRES-Cre mice (JAX, stock 028867), Sst-IRES-Cre mice (JAX, stock 013044), or Vip-IRES-Cre mice (JAX, stock 031628) with Ai14 mice (JAX, stock 007908). Heterozygous Cx3cr1-eGFP(+/-) offspring for microglia analysis were produced by crossing homozygous Cx3cr1-eGFP mice (JAX, stock 005582) with C57Bl/6J mice (JAX, stock 000664). At P6, P10, and P14, the following genetic mouse strains were used: Slc32a1-IRES-Cre mice (JAX, stock 016962) were crossed with Ai65 reporter mice (JAX, stock 021875) and further crossed with Lamp5-P2A-FlpO mice (JAX, stock 037340) to produce triple transgenic offspring for layer 1 (L1) Slc32a1+/Lamp5+ cells. Layer 2/3 (L2/3) Calb2+ cells were labeled by crossing Calb2-IRES-Cre mice (JAX, stock 010774) with Ai14 reporter mice. Layer 4 (L4) Nr5a1+ cells were labeled by crossing Nr5a1-Cre mice (Mutant Mouse Resource & Research Center, stock 036471-UCD) with Ai14 reporter mice. For layer 5 (L5) Rbp4+ cells, Rbp4-Cre KL100 mice (Mutant Mouse Resource & Research Center, stock 037128-UCD) were crossed with Ai14, Ai193 (JAX, stock 034111), or Ai224 reporter mice (JAX, stock 037382). Layer 6 (L6) Ntsr1+ cells were labeled by crossing Ntsr1-Cre GN220 mice (Mutant Mouse Resource & Research Center, stock 030648-UCD) with Ai14 reporter mice. Layer 6b (L6b) Cplx3+ cells were labeled by crossing Cplx3-P2A-FlpO mice (JAX, stock 037338) with Ai193 or Ai227 (JAX, stock 037383) reporter mice. These details are found in Table 1, Supplementary Information, and the Methods section of the paper.

Wild animals

The study did not involve wild animals.

Reporting on sex

The study was designed to include both sexes, male and female, for data collection and analyses. Approximately equal numbers of both sexes make up the overall sample size and the group sample size (per age and per cell type). Exact details can be found in Supplementary Information.

Field-collected samples

The study did not involve samples collected from the field.

Ethics oversight

This study protocol was approved by the Institutional Animal Care and Use Committee (IACUC) at Penn State College of Medicine and the Allen Institute for Brain Science.

Note that full information on the approval of the study protocol must also be provided in the manuscript.

Plants

Seed stocks

Report on the source of all seed stocks or other plant material used. If applicable, state the seed stock centre and catalogue number. If plant specimens were collected from the field, describe the collection location, date and sampling procedures.

Novel plant genotypes

Describe the methods by which all novel plant genotypes were produced. This includes those generated by transgenic approaches, gene editing, chemical/radiation-based mutagenesis and hybridization. For transgenic lines, describe the transformation method, the number of independent lines analyzed and the generation upon which experiments were performed. For gene-edited lines, describe the editor used, the endogenous sequence targeted for editing, the targeting guide RNA sequence (if applicable) and how the editor was applied.

Authentication

Describe any authentication procedures for each seed stock used or novel genotype generated. Describe any experiments used to assess the effect of a mutation and, where applicable, how potential secondary effects (e.g. second site T-DNA insertions, mosiacism, off-target gene editing) were examined.
